# Mdivi-1 and mitochondrial fission: recent insights from fungal pathogens

**DOI:** 10.1007/s00294-019-00942-6

**Published:** 2019-02-19

**Authors:** Barbara Koch, Ana Traven

**Affiliations:** 1 Infection and Immunity Program and the Department of Biochemistry and Molecular Biology, Monash University, Clayton, VIC 3800 Australia; 2 Present Address: Protein, Science and Engineering, Callaghan Innovation, Christchurch, 8140 New Zealand

**Keywords:** Mdivi-1, Mitochondria, Mitochondrial fission, Fungal pathogens, *Candida albicans*, *Cryptococcus neoformans*, *Aspergillus fumigatus*

## Abstract

Mitochondrial fission shows potential as a therapeutic target in non-infectious human diseases. The compound mdivi-1 was identified as a mitochondrial fission inhibitor that acts against the evolutionarily conserved mitochondrial fission GTPase Dnm1/Drp1, and shows promising data in pre-clinical models of human pathologies. Two recent studies, however, found no evidence that mdivi-1 acts as a mitochondrial fission inhibitor and proposed other mechanisms. In mammalian cells, Bordt et al. showed that mdivi-1 inhibits complex I in mitochondria (Dev Cell 40:583, 2017). In a second study, we have recently demonstrated that mdivi-1 does not trigger a mitochondrial morphology change in the human yeast pathogen *Candida albicans*, but impacts on endogenous nitric oxide (NO) levels and inhibits the key virulence property of hyphal formation (Koch et al., Cell Rep 25:2244, 2018). Here we discuss recent insights into mdivi-1’s action in pathogenic fungi and the potential and challenges for repurposing it as an anti-infective. We also outline recent findings on the roles of mitochondrial fission in human and plant fungal pathogens, with the goal of starting the conversation on whether the research field of fungal pathogenesis can benefit from efforts in other disease areas aimed at developing therapeutic inhibitors of mitochondrial division.

## Introduction

Millions of people are affected by fungal infections in the world, including a large number of deaths estimated to surpass a million per year (Brown et al. 2012). Fungal cells are fundamentally very similar to mammalian cells. This is generally thought to create a problem for developing antifungal compounds, while minimizing potential toxicity and adverse effects. Fungal models are used to understand human biology and disease conditions (Botstein and Fink 2011; Hartwell 2004; Krobitsch and Lindquist 2000; Menne et al. 2007; Santos et al. 2018; Sun et al. 2016; van Pel et al. 2013), and the reverse is also possible—in some instances the field of medical mycology might be able to exploit the similarities between fungal and mammalian cells, to build on the knowledge of cellular processes and compounds that are of interest in non-infectious human diseases. One such case is mitochondrial fission, the process by which mitochondria divide.

Mitochondrial fission plays physiological roles in normal cellular functions, promoting distribution of mitochondria between cells during division and elimination of those mitochondria that have been damaged, reviewed in (Friedman and Nunnari 2014; Nunnari and Suomalainen 2012). However, excessive fission, which occurs in response to stressors, during programmed cell death and in human disease pathologies, leads to fragmentation of mitochondria, which in turn causes mitochondrial dysfunction (Ayanga et al. 2016; Cereghetti et al. 2010; Costa et al. 2010; Fannjiang et al. 2004; Frank et al. 2001; Guo et al. 2013; Iqbal and Hood 2014; Lutz et al. 2009; Rambold et al. 2011; Rehman et al. 2012; Song et al. 2011; Wang et al. 2008; Xie et al. 2015). Inhibiting fission of mitochondria could improve mitochondrial and cellular health, and is thought of as a potential therapeutic strategy in neurodegenerative and neuropathological conditions, cardiovascular diseases (stroke, heart attack) and cancer (Brooks et al. 2009; Fannjiang et al. 2004; Gomes et al. 2011; Grohm et al. 2012; Ong et al. 2010; Rambold et al. 2011; Rappold et al. 2014; Sharp et al. 2015; Wang et al. 2017; Xie et al. 2015).

Fungal and mammalian machineries for mitochondrial fission are equivalent, and the main factor which performs mitochondrial fission, the GTPase Dnm1 (also known as Drp1), is conserved. Based on this, can the medical mycology field benefit from efforts aimed at developing mitochondrial fission inhibitors (Cassidy-Stone et al. 2008; Lackner and Nunnari 2010; Mallat et al. 2018; Numadate et al. 2014; Qi et al. 2013; Rosdah et al. 2016)? In other words, would mitochondrial fission inhibitors be of use as antifungals? To try to answer this question, here we discuss recent insights into mitochondrial fission in the main human pathogenic fungi (*Candida albicans, Cryptococcus neoformans* and *Aspergillus fumigatus*), as well as the important plant pathogen *Magnaporthe oryzae* (Chang and Doering 2018; Koch et al. 2018; Neubauer et al. 2015; Zhong et al. 2016). We also discuss our recent work on the putative mitochondrial division inhibitor mdivi-1 in *C. albicans* (Koch et al. 2018). Mdivi-1 was the first specific inhibitor of Dnm1/Drp1 to be discovered (Cassidy-Stone et al. 2008), and follow-on studies indicate therapeutic promise for non-infectious diseases, reviewed in (Rosdah et al. 2016). A complex and somewhat controversial scenario has emerged regarding the mechanism of action of mdivi-1. Two recent studies, ours in *C. albicans* (Koch et al. 2018) and a previous one in mammalian cells (Bordt et al. 2017) cast doubt on the effect of mdivi-1 on mitochondrial morphology and show other metabolic mechanisms to be involved. Nevertheless, mdivi-1 has activity that is of interest in fungal infections. In this mini-review, we will consider its mechanism of action and potential for repurposing as an antifungal agent.

## Mitochondrial fission in pathogenic fungal species

**Fig. 1:**
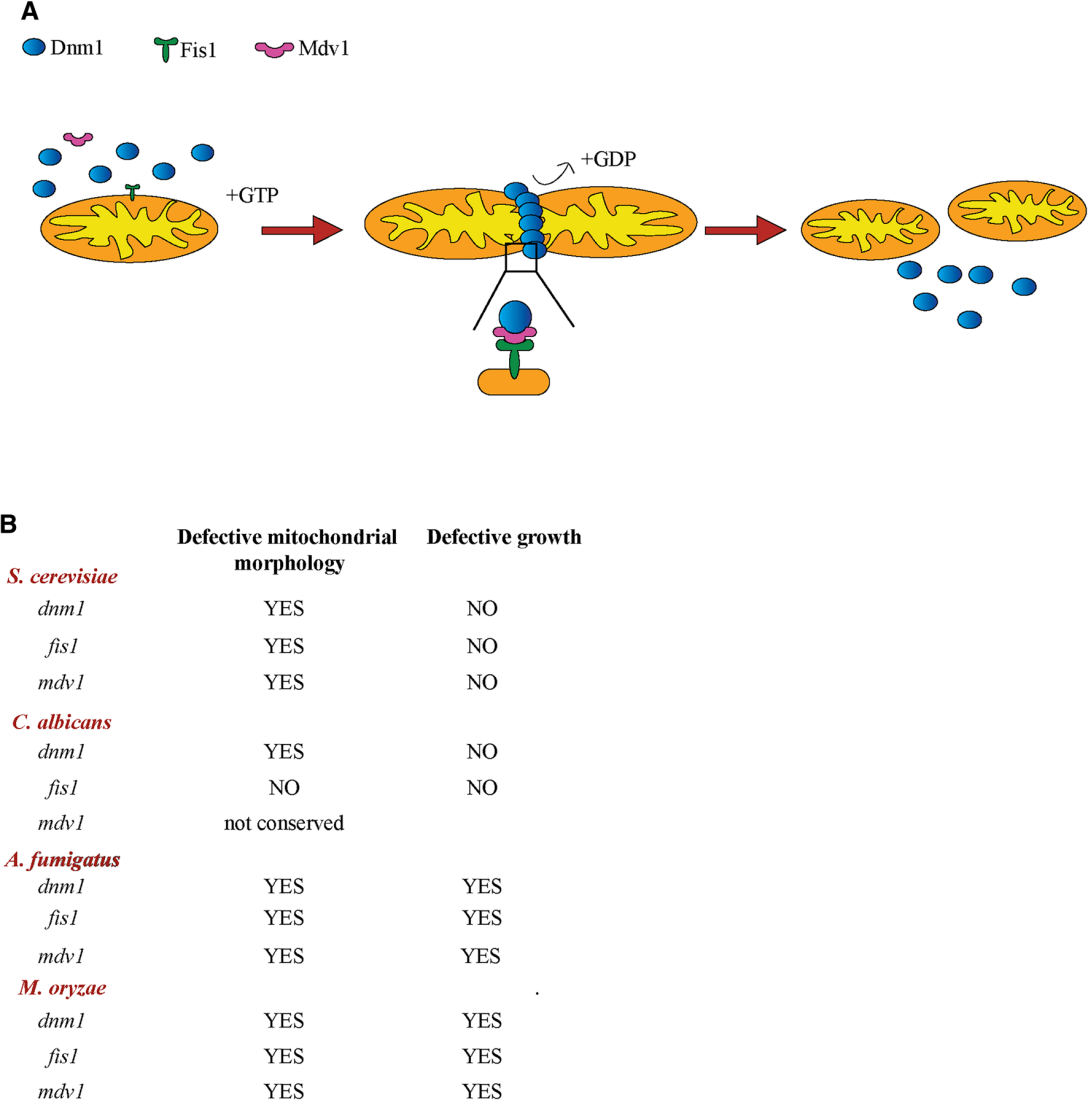
The mitochondrial fission apparatus in fungal species. **a** Cartoon of the mitochondrial fission machinery as known in *S. cerevisiae*. **b** Effects on mitochondrial morphology and growth following inactivation of mitochondrial fission components in fungal species. For those species where we indicate no growth defect, this is under standard and optimal laboratory conditions. Some cellular fitness changes have been reported for the *S. cerevisiae* mutants under specific conditions, but we want to make the point that the general fitness effects of mitochondrial fission mutations are profoundly different between yeast and filamentous fungi. Data for pathogens are based on Chang and Doering (2018), Koch et al. (2018), Neubauer et al. (2015) and Zhong et al. (2016)

Our understanding of the fungal mitochondrial fission apparatus is founded in decades of studies in the model yeast *Saccharomyces cerevisiae* (Fig. 1a) (Labbe et al. 2014). Organelle fission is performed by the Dnm1, which forms rings at the location where mitochondria will divide, and then contracts mitochondria in a process that is coupled to GTP hydrolysis (Bleazard et al. 1999; Ingerman et al. 2005; Mears et al. 2011; Otsuga et al. 1998). Dnm1 is recruited to mitochondria by co-factors: Fis1, a protein in the mitochondrial outer membrane (Mozdy et al. 2000), and Mdv1 that binds to both Fis1 and Dnm1 and brings them together (Cerveny and Jensen 2003; Cerveny et al. 2001; Karren et al. 2005; Tieu and Nunnari 2000; Tieu et al. 2002). *Saccharomyces cerevisiae* deletion mutants in *DNM1, FIS1* or *MDV1* display a mitochondrial fission defect, resulting in the formation of long, hyper-connected mitochondria, and also have some other cellular phenotypes related to fitness, as well as organelle and membrane structure and contacts (Dimmer et al. 2002; Elbaz-Alon et al. 2014; Gorsich and Shaw 2004; Kanki et al. 2009; Prevost et al. 2018; Qian et al. 2012). Deletion of *CAF1*, a paralog of *MDV1*, does not cause a fission defect by itself, but it potentiates the defect of *mdv1* mutants (Griffin et al. 2005).

### The mitochondrial fission apparatus in pathogenic fungi

In all four of the pathogenic fungal species that were recently studied (*C. albicans, C. neoformans, A. fumigatus* and *M. oryzae*), deletion of *DNM1* triggered a mitochondrial morphology defect consistent with reduced fission (Chang and Doering 2018; Koch et al. 2018; Neubauer et al. 2015; Zhong et al. 2016) (Fig. 1b). This was expected, given the high conservation of Dnm1 and its roles in mitochondrial fission in eukaryotes. Deletion of the Dnm1 co-factor *FIS1* caused a mitochondrial fission defect in *C. neoformans, A. fumigatus* and *M. oryzae* (Chang and Doering 2018; Neubauer et al. 2015; Zhong et al. 2016), consistent with studies in *S. cerevisiae* (Griffin et al. 2005; Mozdy et al. 2000). However, somewhat surprisingly, we showed that the *C. albicans fis1* deletion mutant displays normal mitochondrial morphology (Koch et al. 2018) (Fig. 1B). Regarding Mdv1, homologs could be found in *C. neoformans, A. fumigatus* and *M. oryzae* and their inactivation triggered a mitochondrial morphology defect (Chang and Doering 2018; Neubauer et al. 2015; Zhong et al. 2016) (Fig. 1b). *C. albicans* was again different, as its genome does not encode an obvious homolog of Mdv1 (candidagenome.org). Is the mitochondrial fission apparatus in *C. albicans* really all that different to the other fungal species studied? Further work will be needed to address this interesting question. However, we suspect that, rather than a significant departure from the *S. cerevisiae* model, the *C. albicans* Fis1 homolog does have a role in mitochondrial fission, but perhaps loss of its function can be compensated for by another factor. That other factor could be a divergent, but functional homolog of Mdv1 that can be anchored to the outer mitochondrial membrane to bring Dnm1 to the organelle. Our findings in *C. albicans* are paralleled by the situation in mammalian cells. In some mammalian cell types, inactivation of Fis1 does not lead to mitochondrial morphology defects, and several other co-factors in the mitochondrial outer membrane can recruit the Dnm1 homolog Drp1 to mitochondria, reviewed in (Labbe et al. 2014). Also, like in *C. albicans*, no obvious homolog of Mdv1 can be found in mammals (Labbe et al. 2014).

### Impact of mitochondrial fission of cellular growth and fitness of fungal pathogens

Disrupting mitochondrial fission had drastically different effect on growth rates and cellular fitness between yeasts and filamentous fungi. In the yeasts *C. albicans* and *C. neoformans* mitochondrial fission mutants did not show any obvious growth defects in vitro, not even under stressful conditions such as elevated temperature, non-preferred and non-fermentable carbon sources or in response to stressors (Chang and Doering 2018; Koch et al. 2018) and (Koch and Traven unpublished). The *C. neoformans* mitochondrial fission mutants also displayed normal growth in vivo in the lung in the murine inhalation model of cryptococcal infections, and survived normally in innate immune phagocytes (macrophages) in vitro (Chang and Doering 2018). In contrast, in the filamentous pathogens *A. fumigatus* and *M. oryzae* mitochondrial fission mutants showed drastically reduced hyphal growth in vitro (Neubauer et al. 2015; Zhong et al. 2016), and the *M. oryzae* mitochondrial fission mutants were less virulent in a plant infection model (Zhong et al. 2016). Virulence of the *A. fumigatus* mitochondrial fission mutants was only tested in the *Galleria mellonella* larvae model, where they displayed normal virulence (Neubauer et al. 2015). It remains to be seen how they grow and infect a mammalian host. The conclusion that inactivation of mitochondrial fission has a bigger effect on cellular growth in filamentous fungi compared to yeasts is supported by the previous work in model fungal species (Gerstenberger et al. 2012; Mozdy et al. 2000; Otsuga et al. 1998).

### Is mitochondrial fission important for filamentous hyphal growth of yeast species?

**Fig. 2:**
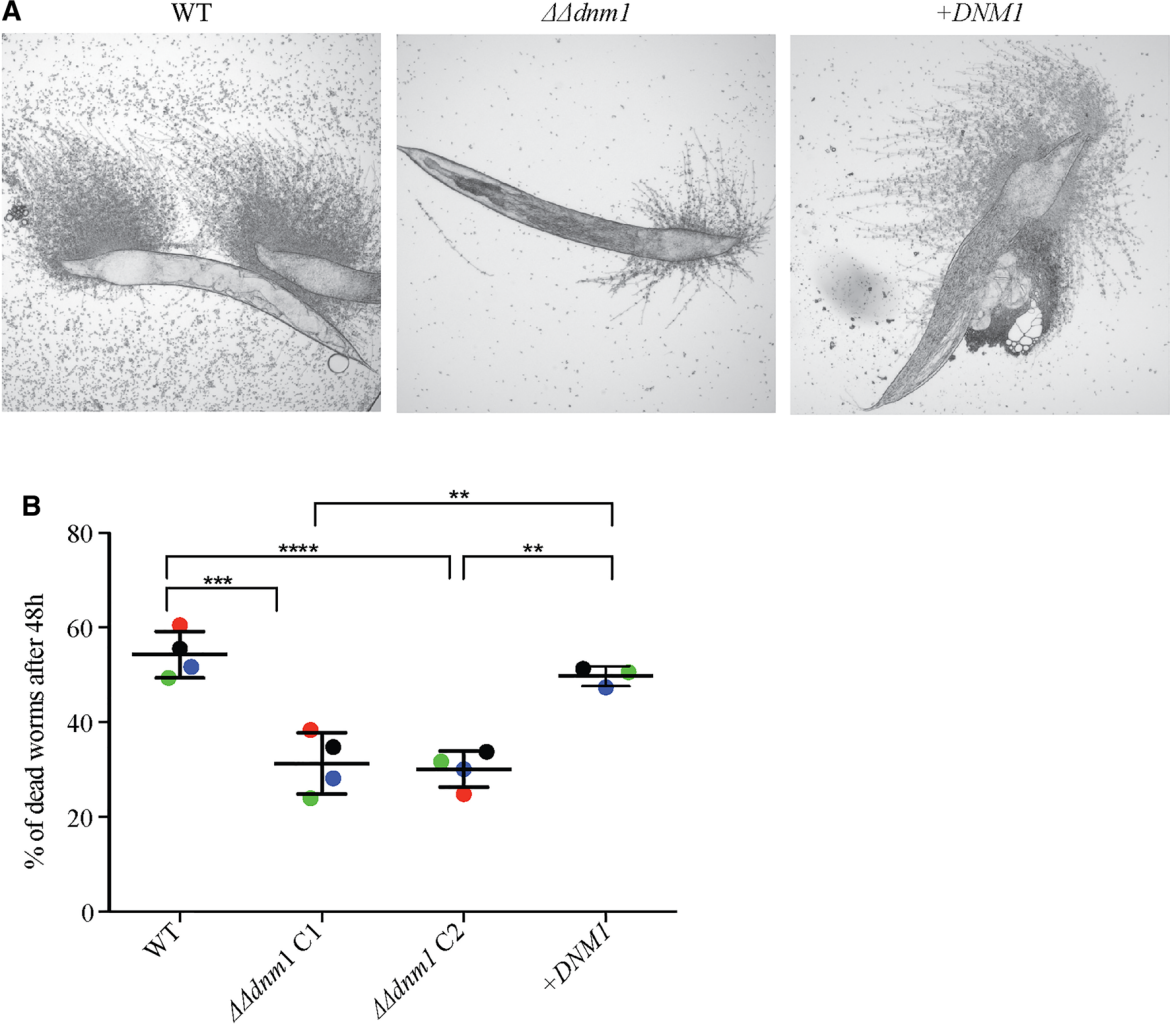
*C. albicans* Dnm1 plays a role in hyphal invasion in the worm infection model. **a** The worm *C. elegans* (*glp-4; sek-1*) was infected with *C. albicans* strains as follows: wild type, a *DNM1* deletion strain *∆∆dnm1* or a complemented mutant strain (+ *DNM1*). The strains are described in (Koch et al. 2018), and the worm *C. albicans* infection method is described in detail in our previous publication (Koch et al. 2017). Shown are representative images after 48 h of infection. **b** The worm infection protocol was performed as in **a** and dead worms killed due to penetrative hyphal growth of *C. albicans* were counted after 48 h. Two independent *∆∆dnm1* strains were used (labeled as C1 and C2). Four biological replicates were done for the wild type and the *∆∆dnm1* strain, while the complemented (+ *DNM1*) strain was assayed in three of the biological replicates. The independent experiments are color coded. Shown are the mean and standard deviation. Statistical significance was determined with one-way ANOVA and Tukey’s multiple comparisons test. **p > 0.001, ***p > 0.0001, ****p < 0.0001

Since mitochondrial fission is important for hyphal growth of the filamentous molds *A. fumigatus* and *M. oryzae*, an interesting question is whether it is necessary for hyphal growth of a yeast species. Our data in *C. albicans* suggest “no”. While *C. albicans* grows in yeast morphology under default conditions, it forms filamentous hyphae in response to a variety of environmental signals. This process of morphology change is linked to pathogenicity (Sudbery 2011). The *C. albicans dnm1* displayed a modest shortening of invasive hyphae on solid medium (plates) (Koch et al. 2018). However, in liquid medium, where there is no resistance to hyphal growth, it formed wild type-looking hyphae (Koch et al. 2018). The shorter hyphae on solid medium suggested to us that perhaps the *dnm1* mutant strain might form hyphae with less invasive capacity. We tested this idea using the worm infection model, because worm killing by *C. albicans* depends on the ability of fungal hyphae to penetrate the animal’s cuticle. Indeed, the *dnm1* mutant was moderately less virulent in the worm model (Fig. 2). Whether reduced hyphal invasion potential of the *C. albicans dnm1* mutant translates to reduced virulence in mice awaits testing.

### So, is inhibiting mitochondrial fission promising as an antifungal strategy?

Based on the growth defects of mitochondrial fission mutants, it could be for filamentous pathogens. It is, however, worth noting that recent work showed that killing of *A. fumigatus* by human immune cells results in mitochondrial fragmentation (Ruf et al. 2018). This would suggest that inhibiting mitochondrial fission might help the pathogen to evade immune responses. Hyper-fused mitochondria have also been previously linked to increase survival of *Cryptococcus gattii* in macrophages (Ma et al. 2009; Voelz et al. 2014). Clearly, much more fundamental knowledge is needed on the roles of mitochondrial fission in fungal pathogenesis before we know if its targeting is warranted for therapy.

## The putative mitochondrial fission inhibitor mdivi-1: what roles does it play in fungal pathogens?

Ten years ago, the lab of Jodi Nunnari reported the discovery of mdivi-1, a compound that triggers a mitochondrial fission defect (Cassidy-Stone et al. 2008). The mechanism is conserved between the model yeast *S. cerevisiae* and mammalian cells, and it involves allosteric inhibition of Dnm1 self-assembly into rings. That Dnm1 is the mdivi-1 target was established with in vitro experiments with the purified yeast protein (assaying for GTPase activity and the effects of mdivi-1 on the formation of Dnm1 spirals using electron microscopy), and also by target overexpression with mammalian Drp1 in cell culture to demonstrate reduced effects of the compound (Cassidy-Stone et al. 2008). A subsequent study used in vitro GTPase activity assays to show that mdivi-1 inhibits mammalian Drp1 (Numadate et al. 2014), and a recent review of the literature concluded that, overall, there is substantial experimental support for the notion that mdivi-1 inhibits mitochondrial fission in mammalian cells (Smith and Gallo 2017). Mdivi-1 has shown therapeutic promise in a range of disease models, including animal studies of cardiovascular dysfunction, brain cancer, and Parkinson’s disease (Brooks et al. 2009; Grohm et al. 2012; Lackner and Nunnari 2010; Ong et al. 2010; Rappold et al. 2014; Rehman et al. 2012; Wang et al. 2017; Xie et al. 2015).

### Mdivi-1 might not always inhibit mitochondrial fission

The notion that the primary cellular target of mdivi-1 is Dnm1 and mitochondrial fission was challenged by two recent reports: (1) we showed that mdivi-1 does not cause a steady state mitochondrial morphology defect in the pathogenic yeast *C. albicans*, and deletion of *DNM1* does not replicate the phenotypic effects of mdivi-1 on *C. albicans* cells (Koch et al. 2018). Our results contrast with the reported inhibition of mitochondrial fission by mdivi-1 in *S. cerevisiae* (Cassidy-Stone et al. 2008). (2) Bordt et al. showed that mdivi-1 does not trigger changes in mitochondrial morphology in mammalian cells and it does not inhibit mammalian Drp1 GTPase activity in vitro (Bordt et al. 2017). Their results contrast with the original study by Cassidy-Stone et al. (2008) and subsequent reports in various mammalian systems, including the aforementioned demonstration of mdivi-1-dependent inhibition of Drp1 in vitro (Numadate et al. 2014). Bordt et al. were, however, able to show that mdivi-1 inhibits *S. cerevisiae* Dnm1 in vitro (Bordt et al. 2017), similarly to the initial report by Cassidy-Stone et al. (2008), opening up the possibility that mdivi-1 has different effects on fungal and mammalian Dnm1. But is *S. cerevisiae* a good model for other fungal species in this case? As mentioned, our study in *C. albicans* showed the opposite results regarding the ability of mdivi-1 to inhibition of mitochondrial fission to what has been shown in *S. cerevisiae* (“yes” in *S. cerevisiae* and “no” in *C. albicans*) (Koch et al. 2018), (Cassidy-Stone et al. 2008). We are aware of only one other fungal species in which the effects of mdivi-1 on mitochondrial morphology were tested. In the filamentous pathogen *M. oryzae* mdivi-1 did trigger a mitochondrial morphology change towards less punctate and more fused structures, consistent with inhibition of organellar fission (Zhong et al. 2016). Why these contrasting effects of mdivi-1 are seen in different systems is not clear at the moment. “Trivial” explanations such as the source of the compound, concentrations used, stock solutions, and solubility in water (mdivi-1 is poorly water-soluble), do not appear to be the reason for the discrepancies in mammalian systems (Smith and Gallo 2017). Regarding fungi, we do not know why *C. albicans* behaves differently to *S. cerevisiae* and *M. oryzae*, but we note the differences in the mitochondrial fission apparatus in *C. albicans* compared to the other fungi, as shown in our recent study (Koch et al. 2018), and discussed above.

### Mdivi-1 is a novel inhibitor of hyphal growth in *C. albicans*

Although it did not affect mitochondrial morphology in *C. albicans*, mdivi-1 inhibited hyphal growth (Koch et al. 2018). Since hyphal formation is an important virulence-related process, we decided to discern the processes affected by mdivi-1 in *C. albicans*. A detailed RNAseq experiments over a time course of 2 h following addition of mdivi-1 to a hyphal culture revealed that transcript levels for genes expressed during hyphal growth were reduced, and there were several changes to metabolism-related gene expression indicative of mitochondrial and metabolic stress (Koch et al. 2018). Genes encoding subunits of the mitochondrial respiratory complexes were transiently inhibited by mdivi-1 and, at the same time expression of alternative oxidases was increased. These results suggested that perhaps mdivi-1 inhibited mitochondrial respiration in some way, which would be consistent with the study of Bordt et al. that reported that the second target of mdivi-1 in mammalian cells is respiratory complex I (Bordt et al. 2017). While we cannot exclude this mechanism, treatment of *C. albicans* with mdivi-1 did not phenocopy complex I mutants with respect to growth phenotypes (Koch et al. 2018). Other transcriptional changes related to metabolism include upregulation of the glyoxylate cycle, gluconeogenesis and fatty acid oxidation, as well as a large upregulation of amino acid biosynthesis-related genes, particularly arginine biosynthesis. Some of the these metabolic changes triggered by mdivi-1 also occur to *C. albicans* following phagocytosis by macrophages (Lorenz et al. 2004; Tucey et al. 2018), indicating that mdivi-1 creates a metabolic situation which is in part similar to the macrophage phagosome. However, *C. albicans* transitions from yeast to hyphae in macrophages, while in the presence of mdivi-1 it cannot transition from yeast to hyphae, and it also cannot maintain hyphal growth when mdivi-1 is added to pre-formed hyphae (Koch et al. 2018). How metabolic stress signals control hyphal formation in these different scenarios remains to be understood.

### Another mdivi-1-dependent cellular pathway discovered in *C. albicans*

In addition to metabolic changes, we showed that mdivi-1 triggered a reduction of endogenous nitric oxide (NO) levels in *C. albicans* cells, and used this discovery to show for the first time that endogenous NO plays an important role in hyphal formation by *C. albicans* (Koch et al. 2018). Our data suggest that the main transcriptional repressor of hyphae-specific genes, Nrg1, is downstream of mdivi-1 and NO-dependent regulation of hyphal gene expression and hyphal growth (Koch et al. 2018). With these data, we established that, in addition to mitochondrial morphology and respiration via complex I inhibition, mdivi-1 also targets NO-dependent signaling in cells. Signaling via NO is an important physiological process in mammalian cells, and it will be interesting to test if mdivi-1 interferes with it in mammalian systems.

## Mdivi-1 as an antifungal agent?

Development of mdivi-1 as human therapeutic agent is of interest, but, as recently reviewed, it is not trivial (Rosdah et al. 2016). Mdivi-1 is poorly soluble in water, and in our study in *C. albicans* we used a fungal strain in which the genes encoding major efflux pumps were deleted (Koch et al. 2018). Comparing this strain with an efflux-competent strain is indicative of mdivi-1 being effluxed. Also, the safety and pharmacological properties of mdivi-1 are still to be characterised in detail (Rosdah et al. 2016).

### Effects of mdivi-1 on fungi in infection

Treatment with mdivi-1 reduced infection of barley leaves with *M. oryzae* (Zhong et al. 2016), showing promising antifungal properties in a plant infection model. Regarding human fungal pathogens, our study in *C. albicans* is to our knowledge the first one to test the effects of mdivi-1 on virulence-related biology. When mdivi-1 was added to *C. albicans* macrophage co-cultures, hyphal formation was represssed and two important hyphae-dependent immune cell processes were compromised: cell death of macrophages caused by *C. albicans* infection was reduced, and macrophages were not inducing maturation of the inflammatory cytokine IL-1β as efficiently as in control conditions (Koch et al. 2018). Mdivi-1 also reduced hyphae that *C. albicans* makes in the worm *C. elegans* (Koch et al. 2018). How mdivi-1 behaves in a mammalian model of fungal infection has not been tested yet. The effects of mdivi-1 in vitro suggest that this compound could modulate levels of inflammation and reduce hyphae-dependent pathogenicity.

### Effects of mdivi-1 on immune cells in infection

If it was to be used as antifungal agent, mdivi-1 would not only target fungal cells, but would also have an effect on host cells. A recent manuscript reported that mdivi-1, when coupled with a mitochondrial fusion-promoting compound M1, is able to drive the formation of more fused mitochondrial network structure in T cells, which had beneficial effects in mouse models of tumorigenesis and infection (Buck et al. 2016). When T cells that were treated *ex vivo* with mdivi-1 plus M1 were transferred into mice, the animals mounted an improved T cell response to the bacterium *Listeria monocytogenes* (Buck et al. 2016). The same study also showed that mdivi-1 reduced the switch to Warburg metabolism (aerobic glycolysis) in murine bone marrow-derived macrophages, which were activated by the bacterial ligand lipopolysaccharide (LPS) and interferon gamma. In the same scenario, mdivi-1 also reduced the expression of Nos2 in macrophages, which is the nitric oxide synthase needed for making NO to kill microbial invaders. A similar effect to reduce aerobic glycolysis was seen in LPS-treated dendritic cells. It is important to note that in both macrophages and dendritic cells, the switch to increased aerobic glycolysis upon encountering LPS was still occurring in a substantial manner in mdivi-1-treated cells, and mdivi-1-treated macrophages expressed Nos2 upon LPS activation above resting levels (Buck et al. 2016). This suggests that mdivi-1 reduces, but does not prevent innate immune activation by LPS.

The switch of innate immune cells to aerobic glycolysis upon activation is a conserved feature and also occurs upon *C. albicans* challenge, where it is important for mounting cytokine responses and survival of mice upon systemic infection (Cheng et al. 2016; Dominguez-Andres et al. 2017). However, we have recently shown that the absolute reliance of macrophages on glycolysis once they switch to Warburg metabolism opens a window of opportunity for *C. albicans*, whereby the pathogen can outcompete macrophages for glucose and kill them in the process (Tucey et al. 2018). Therefore, modulation of glucose homeostasis and immune cell metabolism might require a balance for optimal antimicrobial responses, which mdivi-1 might help to achieve. Reduction of the glycolytic shift in activated immune cells by mdivi-1 might also act to dampen inflammation and prevent hyper-inflammatory pathology in infection. Since mdivi-1 has mostly been studied as a drug lead in non-infectious condition, its potential benefits in infection are highly speculative at the moment. Further work should clarify its effects on fungal pathogens and immune cells in infection.
